# A Cartesian-Based Trajectory Optimization with Jerk Constraints for a Robot

**DOI:** 10.3390/e25040610

**Published:** 2023-04-03

**Authors:** Zhiwei Fan, Kai Jia, Lei Zhang, Fengshan Zou, Zhenjun Du, Mingmin Liu, Yuting Cao, Qiang Zhang

**Affiliations:** 1 State Key Laboratory of Robotics, Shenyang Institute of Automation, Chinese Academy of Sciences, Shenyang 110016, China; 2 Institutes for Robotics and Intelligent Manufacturing, Chinese Academy of Sciences, Shenyang 110169, China; 3 University of Chinese Academy of Sciences, Beijing 100049, China; 4 SIASUN Robot & Automation Co., Ltd., Shenyang 110169, China; 5 School of Automation, Jiangsu University of Science and Technology, No. 666 Changhui Road, Zhenjiang 212100, China

**Keywords:** time-optimal trajectory planning, iterative optimization, jerk limits, time-optimal path parameterization, phase plane

## Abstract

To address the time-optimal trajectory planning (TOTP) problem with joint jerk constraints in a Cartesian coordinate system, we propose a time-optimal path-parameterization (TOPP) algorithm based on nonlinear optimization. The key insight of our approach is the presentation of a comprehensive and effective iterative optimization framework for solving the optimal control problem (OCP) formulation of the TOTP problem in the $\left(s,\dot{s}\right)$-phase plane. In particular, we identify two major difficulties: establishing TOPP in Cartesian space satisfying third-order constraints in joint space, and finding an efficient computational solution to TOPP, which includes nonlinear constraints. Experimental results demonstrate that the proposed method is an effective solution for time-optimal trajectory planning with joint jerk limits, and can be applied to a wide range of robotic systems.

## 1. Introduction

Presently, industrial robotics has a wide range of applications, including welding, palletizing, grinding and polishing, assembly, and painting [1,2,3]. After decades of research, the problem of time-optimal trajectory planning (TOTP) of robots along specified paths has been extensively studied to optimize operation time and improve the efficiency of automated industrial robot operations [4]. TOTP is based on interpolation and introduces the concepts of constraint and optimization to maximize the performance of the robot and ensure the shortest time, while making the trajectory smooth and the operation run smoothly [5]. Time-optimal path parameterization (TOPP) is a fast method for determining critical conditions for navigating a pre-defined smooth path in a robot system’s configuration space while respecting physical constraints [6]. Although finding the time-optimal parameterization of a path subject to second-order constraints is a well-studied problem in robotics, TOPP subject to third-order constraints (such as jerk and torque rate) has received relatively little attention and remains largely open. Moreover, joint space trajectory planning cannot visualize the end position of the robotic arm, and Cartesian space trajectory planning is often used in many specific industrial scenarios such as welding, cutting, or machining that require operation on a predetermined path. Therefore, a TOTP algorithm that satisfies the joint third-order constraints in Cartesian space is urgently needed.

### 1.1. Related Works

Over the years, many academics have worked on the issue of TOTP for industrial robots. This problem can be roughly divided into three main families of methods: Numerical Integration (NI), Convex Optimization (CO), and Dynamic Programming (DP).

The NI-based strategy was initiated by Bobrow et al. [7] and further developed by other researchers. Kunz et al. [8] provided a circular-blends route differentiability approach to ensure that the trajectory precisely follows the specified path of differentiable joint space. Pham [9] provided a comprehensive solution to the problem of dynamic singularities. Pham et al. [10] proposed TOPP3, a novel TOPP algorithm that addresses third-order constraints, as well as the problem of singularities that may hinder the integration of motion profiles and the smooth connection of optimal profiles.Shen et al. [11,12] proposed various new characteristics of the NI method for TOTP along the defined path, and provided explicit mathematical confirmation of these traits. Lu et al. [13] proposed a time-optimal motion planning method for sculpted surface robot machining that takes joint space and tool tip motion constraints into account. They solved the time-optimal tool motion planning in robot machining using an efficient numerical integration method based on the Pontryagin maximum principle. Methods based on NI explicitly calculate the optimal control at each position along the path, instead of performing an implicit search such as the CO-based method, which makes them very fast. However, finding the switch points between the acceleration and deceleration phases is necessary, and the main reason for their failure.

The CO-based strategy has been expanded upon by numerous researchers after being introduced by Verscheure et al. [14]. Xiao et al. [15] used the cubic polynomial fitting method to construct the maximum pseudo-speed curve that meets the torque and speed limits. Debrouwere et al. [16] proposed an effective sequential convex programming (SCP) method to solve the corresponding nonconvex optimal control problems as a difference of convex (DC) function. Pham et al. [6] presented a TOPP approach based on reachability analysis (TOPP-RA), which iteratively computes the reachable and controllable sets at discrete points along the path by solving linear programming problems (LP). Nagy et al. [17] considered kinematics and dynamics constraints and generated the time-optimal velocity distribution for the LP control problem using the sequential optimization method. Ma et al. [18] converted a nonconvex jerk limit into a linear acceleration constraint and indirectly introduced it into CO for TOTP. This method preserves CO’s convexity and does not increase the number of optimization variables, resulting in a quick calculation speed. CO-based methods are easy to implement and quite robust, and can consider multiple optimization objectives beyond just time. However, the optimization problem they solve is very large. The number of variables and constraint inequalities scales with the discretization grid size, resulting in implementation that is an order of magnitude slower than the NI-based method.

The DP-based approach was first developed by [19] and has since been expanded and improved upon by numerous researchers. Kaserer et al. [20] proposed a DP-based method for solving the optimal path-tracking problem, which uses interpolation in the phase plane. This approach considers joint speed, acceleration, torque, and mechanical power, as well as joint jerk and torque rate limitations. Kaserer et al. [21] extended this method to solve the time-optimal path-tracking problem for cooperative grasping tasks involving two robots, while also accounting for robot speed, acceleration, jerk, and torque constraints. Barnett et al. [22] introduced the bisection algorithm (BA), a novel technique that extends DP approaches to tackle more complex problems with a larger number of constraints. These approaches, which break down the larger problem into smaller sub-problems, become increasingly advantageous as the number of constraints grows, compared to direct transcription methods. Methods based on DP are simple to implement and do not suffer from local minima problems, and they traverse all states at each path point (rather than requiring convex space or convex function assumptions such as the CO-based method). However, the state space to be searched is huge, resulting in implementation being one (or even more) orders of magnitude slower than the CO-based method. Additionally, the DP method cannot truly achieve the global optimal point due to the issue of grid precision.

There are several alternative approaches to the TOTP problem beyond the three groups mentioned above [23,24,25,26,27,28]. Nevertheless, these approaches also neglect the third-order constraint and do not perform planning in Cartesian space. Table 1 summarizes and compares the similarities and differences of the above three methods in the following five aspects: the requirement for calculating switching points, the ability to consider multiple optimization objectives, the ability to achieve the optimal point (rather than approximately achieving the optimal point), the planning space, and the highest constraint order.

### 1.2. Motivations and Contributions

Motivated by previous approaches, this paper proposes aTOTP algorithm that considers joint third-order limits in a Cartesian coordinate system, maximizing the robot operation efficiency while maintaining smoothness and minimizing time. To achieve this, kinematic feasibility is ensured by introducing joint velocity, acceleration, and jerk constraints on the path parameters *s*, which are then relocated to the Cartesian space using a constraint transfer method based on Lie theory (We use the Lie group SE(3) to represent the motion of the robot end-effector in Cartesian space. The detailed description of using Lie theory for robot forward and inverse kinematic analysis and the Jacobian matrix derivation process is presented in the Appendix A), reducing the number of decision variables. After establishing the optimal control problem (OCP) formulation of the TOTP problem in the $\left(s,\dot{s}\right)$ phase plane, the TOPP-RA algorithm is extended to the Cartesian space to obtain an initial solution, and a constraint relaxation approach is used to simplify nonconvex state-update constraints. The method is validated through simulation experiments on a ROS-based platform and real-world experiments on an actual robot, demonstrating effectiveness, generality, and robustness. This paper makes several contributions to the field of optimal trajectory generation:

- A comprehensive and effective framework for iterative optimization is presented to establish the OCP formulation of the TOTP problem, which is described by the path parameter *s*;
- Given an efficient computational solution for computing the nonlinear TOPP in Cartesian space while satisfying third-order constraints in joint space;
- Experiments have demonstrated that the proposed method can effectively generate smoother trajectories that satisfy jerk constraints on a wide range of robot systems.

The remainder of this paper is organized as follows. Section 2 outlines the key features of the OCP model used for the TOPP algorithm. In Section 3, we present the Cartesian-based TOPP-RA method and describe the proposed TOPP algorithm based on iterative optimization. Section 4 reports extensive experimental results. Finally, in Section 5, we provide concluding remarks.

## 2. Problem Statement

In this section, we establish the TOPP problem as an OCP in Cartesian space, which includes joint third-order constraints and an objective function in the $\left(s,\dot{s}\right)$ phase plane. The details of these constraints will be formulated in the following subsections.

### 2.1. General Description

In a *n*-dof robot system, the state profiles in configuration space are denoted by $x\left(t\right)=[q\left(t\right);\dot{q}\left(t\right);\ddot{q}\left(t\right)]$, where $q\in R^{n}$ represents the configuration of the system. The control inputs u(t) represent the third derivative of the joint angles, $\dddot{q}\left(t\right)$, in configuration space. The following is a standard OCP that can be used to describe the time-optimal speed planning problem [29]:

(1) $$\begin{array}{cc} \min & \;J(x(t),u(t))\\ s.t. & \;\dot{x}\left(t\right)=f_{Status-update}\left(x\left(t\right),u\left(t\right)\right),\\ \; & \;x_{min}\leq x\left(t\right)\leq x_{max},u_{min}\leq u\left(t\right)\leq u_{max},t\in \left[0,T\right];\\ \; & \;x\left(0\right)=x_{init},u\left(0\right)=u_{init},x\left(T\right)=x_{goal},u\left(T\right)=u_{goal}. \end{array}$$

$f_{Status-update}=0$ forms the status-update process. $\left[x_{min},x_{max},u_{min},u_{max}\right]$ describes the allowable regions of state and control profiles. $\left[x_{init},u_{init},x_{goal},u_{goal}\right]$ denotes the start and end conditions of the state and control profiles. *T* represents the total time, which is unidentified now.

To translate the above model to a TOPP problem in the $\left(s,\dot{s}\right)$ phase plane, we propose a function $p{\left(s\right)}_{s\in [0,s_{end}]}$ that represents a geometric path in the Cartesian space, and is piece-wise $C^{2}$-continuous. We introduce a time parameterization that itself represents the parameter of the path, as a piece-wise $C^{2}$, increasing scalar function $s:\left[0,T\right]\to \left[0,s_{end}\right]$. The trajectory is then recovered as $p{\left(s\left(t\right)\right)}_{t\in [0,T]}$ [30]. In the rest of this section, we introduce how to complete the transformation of the TOPP problem through $s:\left[0,T\right]\to \left[0,s_{end}\right]$.

### 2.2. Objective Function

To minimize the total time of robot movement, the objective function J(x(t),u(t)) is defined as

(2) $$J=T=\int_{t=0}^{T}1dt$$

Replace the previous equation with ds/ds=1 and change the integral limits from [0,T] (time) to $\left[0,s_{end}\right]$ (*s*) [31]. Formula (2) is updated as follows:

(3) $$J=\int_{t=0}^{T}1dt=\int_{t=0}^{T}\frac{ds}{ds}dt=\int_{s=0}^{s_{end}}\frac{dt}{ds}ds=\int_{s=0}^{s_{end}}\frac{1}{\dot{s}}ds$$

Therefore, to minimize the time, ${\dot{s}}^{-1}\left(s\right)$ should be as small as possible. In other words, $\dot{s}\left(s\right)$ must be as large as possible while still satisfying the various constraints mentioned later. This means that the state trajectory must follow the boundary of the phase diagram plotted by $\left(s,\dot{s}\right)$, which is naturally aligned with TOPP-RA method.

### 2.3. Constraints

In a TOPP problem, there are generally three types of constraints: status-update constraints, constraints on the states/control profiles, and two-point boundary constraints [32].

#### 2.3.1. Status-Update Constraints

The state-update/kinematic constraints of a robot describe the kinematic feasibility of the robot’s motion. Using forward and inverse kinematics, the configuration q in the joint space can be converted to the corresponding Cartesian space representation p (see the Appendix A for transformation method). As a result, the state and control profiles can be expressed in terms of the geometric path p(s), which can then be further transformed to a form represented by path parameters *s*, as shown in Equation (4).

(4) $$\frac{d}{ds}\left[\begin{array}{c} \dot{s}\\ \ddot{s} \end{array}\right]=\left[\begin{array}{c} \frac{\ddot{s}}{\dot{s}}\\ \frac{\dddot{s}}{\dot{s}} \end{array}\right],s\in \left[0,s_{end}\right]$$

The status-update function can be rewritten by performing a second-order Taylor series expansion at $s_{i}$.

(5) $$\begin{array}{c} \dot{s}={\dot{s}}_{i}+\frac{{\ddot{s}}_{i}}{{\dot{s}}_{i}}\Delta \left(s\right)+\frac{d^{2}\dot{s}}{ds^{2}}{|}_{s=\xi }{\left(\Delta \left(s\right)\right)}^{2}\\ \ddot{s}={\ddot{s}}_{i}+\frac{{\dddot{s}}_{i}}{{\dot{s}}_{i}}\Delta \left(s\right)+\frac{d^{2}\ddot{s}}{ds^{2}}{|}_{s=\eta }{\left(\Delta \left(s\right)\right)}^{2} \end{array}$$

where $s\in \left[s_{i},s_{i+1}\right],\xi ,\eta \in \left[s_{i},s\right]\;\mathrm{and}\;\Delta \left(s\right)=s-s_{i}$. Let us define the first-order status-update discretization function as follows:

(6) $$\begin{array}{c} \dot{s}={\dot{s}}_{i}+\frac{{\ddot{s}}_{i}}{{\dot{s}}_{i}}\Delta \left(s\right)\\ \ddot{s}={\ddot{s}}_{i}+\frac{{\dddot{s}}_{i}}{{\dot{s}}_{i}}\Delta \left(s\right) \end{array}$$

The error of the first-order status-update discretization function, denoted by $e_{first}^{state}$, is as follows:

(7) $$e_{first}^{state}=O\left(\Delta^{2}\left(s\right)\right)$$

Similarly, by performing a third-order Tylor series expansion at $s_{i}$, the second-order status-update discretization function and its error can be, respectively, rewritten as:

(8) $$\begin{array}{c} \dot{s}={\dot{s}}_{i}+\frac{{\ddot{s}}_{i}}{{\dot{s}}_{i}}\Delta \left(s\right)+\left(\frac{{\dddot{s}}_{i}}{{\dot{s}}_{i}^{2}}-\frac{{\ddot{s}}_{i}^{2}}{{\dot{s}}_{i}^{3}}\right)\Delta^{2}\left(s\right)\\ \ddot{s}={\ddot{s}}_{i}+\frac{{\dddot{s}}_{i}}{{\dot{s}}_{i}}\Delta \left(s\right)-\frac{{\ddot{s}}_{i}{\dddot{s}}_{i}}{{\dot{s}}_{i}^{3}}\Delta^{2}\left(s\right) \end{array}$$

(9) $$e_{second}^{state}=O\left(\Delta^{3}\left(s\right)\right)$$

#### 2.3.2. States/Control Profiles Constraints

The state/control constraints of a robot refer to the physical constraints that the robot must adhere to during its motion process. Typically, these constraints involve the robot’s state variables, such as position, velocity, acceleration, joint angles, and so on. The constraints on the robot’s states and control profiles can be formulated as $x_{min}\leq x\left(t\right)\leq x_{max}$ and $u_{min}\leq u\left(t\right)\leq u_{max}$, respectively, where t∈[0,T]. These constraints essentially limit the speed, acceleration, and jerk of the robot’s joints [33], as illustrated in the following equations.

(10) $$\left[\begin{array}{c} {\dot{q}}_{min}\\ {\ddot{q}}_{min}\\ {\dddot{q}}_{min} \end{array}\right]\leq \left[\begin{array}{c} \dot{q}\left(t\right)\\ \ddot{q}\left(t\right)\\ \dddot{q}\left(t\right) \end{array}\right]\leq \left[\begin{array}{c} {\dot{q}}_{max}\\ {\ddot{q}}_{max}\\ {\dddot{q}}_{max} \end{array}\right],t\in \left[0,T\right]$$

The derivatives of the joints are projected into Cartesian space through the Jacobian matrix, as shown in Formula (11), which yields the derivatives of the path parameter *s*.

(11) $$\begin{array}{cc} J\dot{q} & =p^{\prime }\dot{s}\\ J\ddot{q}+\dot{J}\dot{q} & =p^{\prime \prime }{\dot{s}}^{2}+q^{\prime }\ddot{s}\\ J\dddot{q}+2\dot{J}\dot{q}+\ddot{J}\dot{q} & =p^{\prime \prime \prime }{\dot{s}}^{3}+3p^{\prime \prime }\dot{s}\ddot{s}+p^{\prime }\dddot{s} \end{array}$$

where ${□}^{\prime }$ is defined as the differentiation of □ with respect to the path parameter *s*. Henceforth, we shall refer to $s,\dot{s},\ddot{s},$ and $\dddot{s}$ as the position, velocity, acceleration, and jerk, respectively. By substituting Equation (11) into Equation (10), the inequality constraints on the states/control profiles can be expressed as follows:

(12) $$\left[\begin{array}{c} {\dot{q}}_{min}\\ {\ddot{q}}_{min}\\ {\dddot{q}}_{min} \end{array}\right]\leq \left[\begin{array}{c} a\left(s\right)\dot{s}\\ b\left(s\right){\dot{s}}^{2}+c\left(s\right)\ddot{s}\\ d\left(s\right){\dot{s}}^{3}+e\left(s\right)\dot{s}\ddot{s}+f\left(s\right)\dddot{s} \end{array}\right]\leq \left[\begin{array}{c} {\dot{q}}_{max}\\ {\ddot{q}}_{max}\\ {\dddot{q}}_{max} \end{array}\right],s\in \left[0,s_{end}\right],\mathrm{where}$$

(13) $$\begin{array}{cc} a(s) & :=J^{-1}\left(s\right)p^{\prime }\left(s\right),\\ b(s) & :=J^{-1}\left(s\right)\left(p^{\prime \prime }\left(s\right)-J^{\prime }\left(s\right)J^{-1}\left(s\right)p^{\prime }\left(s\right)\right),\\ c(s) & :=J^{-1}\left(s\right)p^{\prime }\left(s\right),\\ d(s) & :=J^{-1}\left[p^{\prime \prime \prime }\left(s\right)-2J^{\prime }\left(s\right)J^{-1}\left(s\right)\left(p^{\prime \prime }\left(s\right)-J^{\prime }\left(s\right)J^{-1}\left(s\right)p^{\prime }\left(s\right)\right)-J^{\prime \prime }\left(s\right)J^{-1}\left(s\right)p^{\prime }\left(s\right)\right],\\ e(s) & :=3J^{-1}\left(s\right)\left(p^{\prime \prime }\left(s\right)-J^{\prime }\left(s\right)J^{-1}\left(s\right)p^{\prime }\left(s\right)\right),\\ f(s) & :=J^{-1}\left(s\right)p^{\prime }\left(s\right). \end{array}$$

The formulas for calculating each order derivative of the Jacobian matrix ($J^{\prime },J^{\prime \prime }$) will be presented in the Appendix A.

#### 2.3.3. Boundary Constraints

Boundary constraints refer to the limitations imposed on the state and control variables of a robot during the initial and final stages of its operation. The constraints $x\left(0\right)=x_{init}$, $u\left(0\right)=u_{init}$, $x\left(T\right)=x_{goal}$, and $u\left(T\right)=u_{goal}$ define the boundary conditions. These boundary conditions ensure that the state and control profiles at the start moment s=0(t=0) and the end moment $s=s_{end}\left(t=T\right)$ represent the necessary facts at those moments, respectively.

(14) $$\begin{array}{cc} \left[\dot{s}\left(0\right),\ddot{s}\left(0\right),\dddot{s}\left(0\right)\right] & =[{\dot{s}}_{0},{\ddot{s}}_{0},{\dddot{s}}_{0}],\\ \dot{s}\left(s_{end}\right),\ddot{s}\left(s_{end}\right),\dddot{s}\left(s_{end}\right)] & =[{\dot{s}}_{s_{end}},{\ddot{s}}_{s_{end}},{\dddot{s}}_{s_{end}}]. \end{array}$$

In particular, more degrees of freedom are allowed in setting the control profile $\dddot{s}$ at s=0(t=0) to ensure the normal operation of the motor.

As a summary of this section, the following OCP is established to represent the TOPP problem based on Cartesian space:

(15) $$\begin{array}{cc} \min & \;(3)\\ s.t. & \;\;\text{Status-update}\;\mathrm{constraints}\;(4);\\ \; & \;\mathrm{States}/\mathrm{Control}\;\mathrm{profiles}\;\mathrm{constraints}\;(12)\;\mathrm{and}\;(13);\\ \; & \;\text{Two-point}\;\mathrm{boundary}\;\mathrm{constraints}\;(14). \end{array}$$

In general, when moving from the initial state to the target state along a predetermined path, speed planning aims to resolve any potential conflicts that may arise between kinematics-based constraints and environmental constraints. However, due to the nonlinear relationship between the state and control variables, an appropriate initial solution is required to solve the OCP (15). There is a problem with the state/control constraints (12) in OCP (15) because the jerk of the robot is not taken into account when solving the initial solution, which can easily lead to leaving out the free space required for kinematic feasibility. Therefore, directly solving OCP (15) may not always be effective. An alternative option we propose is to build an iterative framework in which the kinematic feasibility is adaptively adjusted when it is found to be inappropriate. The details on how to find an effective computational solution to TOPP with nonlinear constraints are described in Section 3.

## 3. TOPP by Iterative Optimization (TOPP-IO)

This section introduces our proposed Cartesian-based TOPP-IO method. First, we present the initial guess and control group generated by the Cartesian-based TOPP-RA method, followed by an explanation of the principle of the TOPP-IO method.

### 3.1. Cartesian-Based TOPP-RA Method

Combining with [6], we expanded the TOPP-RA method from joint space to Cartesian space, which we call the Cartesian-based TOPP-RA method. The geometric path in Cartesian space, denoted by p(s), is divided into *N* segments with N+1 grid points, where $\left(s_{i},{\dot{s}}_{i},{\ddot{s}}_{i},{\dddot{s}}_{i}\right)$ represents the *i*-th stage state and control profiles, with i∈[0,1,⋯,N]. The constraints of joint acceleration can be formulated as follows, by taking into account (12) and (13):

(16) $$B{\dot{s}}^{2}+C\ddot{s}\leq \ddot{Q}$$

where $B=\left[\begin{array}{c} b(s)\\ -b(s) \end{array}\right],C=\left[\begin{array}{c} c(s)\\ -c(s) \end{array}\right]$ and $\ddot{Q}=\left[\begin{array}{c} {\ddot{q}}_{max}\\ -{\ddot{q}}_{min} \end{array}\right]$. The velocity constraints of the joints are expressed as a range of *i*-stage state variables, $X_{i}=\left[{\left({\dot{s}}_{i}^{2}\right)}^{lower},{\left({\dot{s}}_{i}^{2}\right)}^{upper}\right]$, which reflects the allowable velocity of the joints.

(17) $$\begin{array}{cc} {\left({\dot{s}}_{i}^{2}\right)}^{lower}= & \max_{j}\left\{\frac{{\dot{q}}_{min,j}}{a_{j}\left(s_{i}\right)}|a_{j}\left(s_{i}\right)>0\;\mathrm{or}\;\frac{{\dot{q}}_{max,j}}{a_{j}\left(s_{i}\right)}|a_{j}\left(s_{i}\right)<0\right\},\\ {\left({\dot{s}}_{i}^{2}\right)}^{upper}= & \min_{j}\left\{\frac{{\dot{q}}_{max,j}}{a_{j}\left(s_{i}\right)}|a_{j}\left(s_{i}\right)>0\;\mathrm{or}\;\frac{{\dot{q}}_{min,j}}{a_{j}\left(s_{i}\right)}|a_{j}\left(s_{i}\right)<0\right\}. \end{array}$$

where *j* is the *j*-th element of $a\left(s_{i}\right),{\dot{q}}_{min}$ and ${\dot{q}}_{max}$. The state-update function for constant acceleration over $\left[s_{i},s_{i+1}\right]$ is given by:

(18) $${\dot{s}}_{i+1}^{2}={\dot{s}}_{i}^{2}+2\Delta_{i}{\ddot{s}}_{i}$$

where $\Delta =s_{i+1}-s_{i}$.

#### 3.1.1. Backward Pass

In considering the segment $\left[s_{i},s_{i+1}\right]$ and assuming that the i+1-th feasible range, $S_{i+1}$, is known, the *i*-th feasible range, $S_{i}=\left[{\left({\dot{s}}_{i}^{2}\right)}^{-},{\left({\dot{s}}_{i}^{2}\right)}^{+}\right]$, can be calculated using the following formula:

(19) $$\begin{array}{cc} {\left({\dot{s}}_{i}^{2}\right)}^{-} & :=\min{\dot{s}}_{i}^{2},\\ {\left({\dot{s}}_{i}^{2}\right)}^{+} & :=\max{\dot{s}}_{i}^{2},\\ s.t.\; & {\dot{s}}_{i}^{2}\in X_{i},\\ \; & {\dot{s}}_{i}^{2}+2\Delta_{i}{\ddot{s}}_{i}\in S_{i+1},\\ \; & B{\dot{s}}_{i}^{2}+C{\ddot{s}}_{i}\leq \ddot{Q}. \end{array}$$

Obviously, Formula (19) indicates that for any ${\dot{s}}_{i}^{2}\in S_{i}$, there always exists a state ${\dot{s}}_{i+1}^{2}\in S_{i+1}$ that corresponds to it. In other words, we can always move from the feasible range $S_{i}$ to $S_{i+1}$ using the state-update function. By applying Formula (19) recursively, we can obtain a set of transitive feasible ranges, $\left[S_{0},S_{1},\cdots ,S_{n}\right]$. Any state that belongs to the transitive feasible ranges can be transferred to the ending state when the last feasible range set is determined.

#### 3.1.2. Forward Pass

By transferring ${\dot{s}}_{i}^{2}\in S_{i}$ from step *i* to ${\dot{s}}_{i+1}^{2}\in S_{i+1}$ of step i+1, we can recursively reach the final state $S_{n}$. Furthermore, literature [6] has demonstrated that the transition process occurs on a convex polygon. Therefore, selecting control variables that can reach the upper limit of the next S will result in the shortest task time. This selection exhibits locally greedy behavior while globally optimizing performance. Once the transitive feasible ranges have been derived from the backward pass, the method for transferring ${\left({\dot{s}}_{i}^{2}\right)}^{*}$ to ${\left({\dot{s}}_{i+1}^{2}\right)}^{*}$ using a greedy algorithm is as follows:

(20) $$\begin{array}{cc} {\left({\dot{s}}_{i+1}^{2}\right)}^{*} & :=\max{\left({\dot{s}}_{i}^{2}\right)}^{*}+2\Delta_{i}{\ddot{s}}_{i},\\ s.t.\; & {\left({\dot{s}}_{i}^{2}\right)}^{*}+2\Delta_{i}{\ddot{s}}_{i}\in S_{i+1},\\ \; & B{\left({\dot{s}}_{i}^{2}\right)}^{*}+C{\ddot{s}}_{i}\leq \ddot{Q}. \end{array}$$

where ${\left({\dot{s}}_{i}^{2}\right)}^{*}$ denotes the optimal solution at the *i*-th grid point. By setting deterministic values of ${\left({\dot{s}}_{0}^{2}\right)}^{*}\in S_{0}$ and $S_{n}=\left\{{\left({\dot{s}}_{n}^{2}\right)}^{*}\right\}$, the solution of Cartesian-based TOPP-RA, $[{\left({\dot{s}}_{0}^{2}\right)}^{*},{\left({\dot{s}}_{1}^{2}\right)}^{*},$$\cdots ,{\left({\dot{s}}_{n}^{2}\right)}^{*}]$, is obtained by recursively applying Formula (20).

### 3.2. Principle of the Proposed TOPP-IO Method

The general principle of the TOPP iterative optimization method is illustrated by the pseudo-codes in Algorithm 1. Given a path P in Cartesian space, Algorithm 1 first generates an initial conjecture using the ToppraGuess() function to numerically solve (15) without joint jerk limits. This initial conjecture includes the path discretization, all the parameters required to solve (15), and the initial values of all the decision variables. Then, using the full content of this initial conjecture, Algorithm 1 establishes an iterative OCP where an intermediate optimal solution is obtained from each iteration. After the first three lines of initialization, the while loop is applied to iteratively solve the ${\mathrm{TOPP}}_{OCP}$. Similar to (15), the only difference is that we add (4) as a soft constraint to the objective function. Specifically, this iterative OCP solves the following optimization problems.

(21) $$\begin{array}{cc} \min & \;\left(3\right)+\omega_{soft}\cdot f_{soft}\left(s\right)\\ s.t. & \;\mathrm{States}/\mathrm{Control}\;\mathrm{profiles}\;\mathrm{constraints}\;(12)\;\mathrm{and}\;(13);\\ \; & \;\text{Two-point}\;\mathrm{boundary}\;\mathrm{constraints}\;(14). \end{array}$$

where $\omega_{sift}>0$ is a parameter used to weight the softening of the state updating, and $f_{soft}\left(s\right)$ is denoted as

(22) $$f_{soft}\left(s\right)=\int_{s=0}^{s_{end}}{∥\left[\begin{array}{c} \dot{s}\\ \ddot{s} \end{array}\right]-f_{Status-update}\left(s\right)∥}^{2}ds$$

In each iteration of the while loop, the function $\mathrm{SolveIteratively}({\mathrm{OCP}}_{TOPP},G)$ is used to solve (21) using the initial conjecture G. The function StateUpdateInfeasibility(G) evaluates the infeasibility degree of the status update determined by $f_{soft}\left(s\right)$ as given in (22). When $f_{soft}\left(s\right)$ becomes small enough, i.e., close to $0^{+}$, the function GetTrajectoryInformation(G) is called to extract information about the optimal trajectory from the solution G.
**Algorithm 1:** An Iterative Optimal Method for TOPP **Input**: Geometric path in Cartesian space P **Output**: Optimal trajectory information ${\mathrm{info}}_{opti}$
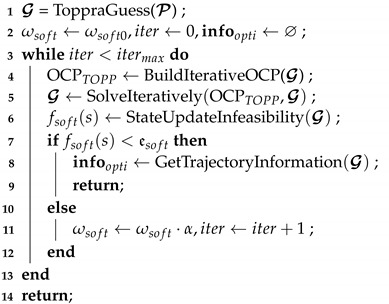


### 3.3. Properties Discussion of Algorithm 1

This subsection describes the relevant properties of the proposed TOPP-IO method in Algorithm 1.

First, the iterative process progressively increases the feasibility and optimality of the phase state. It is assumed that the initial solution obtained by the Cartesian frame TOPP-RA does not satisfy the jerk constraint and, hence, is not status-update feasible. In such cases, restoring status-update feasibility becomes the primary goal of minimizing the objective function of ${\mathrm{OCP}}_{TOPP}$. Therefore, the optimal solution differs from the initial guess by reducing the status-update infeasibility. Although the status-update infeasibility may not be eliminated, the resulting $\left(s,\dot{s}\right)$ phase diagram is closer to being feasible, providing opportunities for further improvement in succeeding iterations.

Second, optimality is achieved when Algorithm 1 exits from line 9. As the iteration continues, the status-update infeasibility approaches $0^{+}$ and incrementing $\omega_{soft}$ expedites the procedure. When the degree of status-update infeasibility is small, the total time (3) in the objective function of (21) dominates. Thus, the objective function of (21) is minimized, closing in on minimizing the original objective function (3) to an accuracy level of $e_{soft}$.

Third, the ${\mathrm{OCP}}_{TOPP}$ is always feasible, which is a crucial cornerstone of the entire iterative framework. With strict restrictions on CPU runtime and a willingness to accept suboptimal solutions, a feasible solution can be obtained at any point by interrupting the iterative optimization process. With very slow motion always feasible, the solution procedure for each (21) is consistently in the feasible region of the solution space when the initial solution is set to 0. Thus, as long as the obtained $\left(s,\dot{s}\right)$ phase diagram’s near-future period is status-update feasible, the resulting phase states can be transferred to the next iterative ${\mathrm{OCP}}_{TOPP}$ for further enhancements.

## 4. Simulation and Real-World Experiment Results

**Figure 1:**
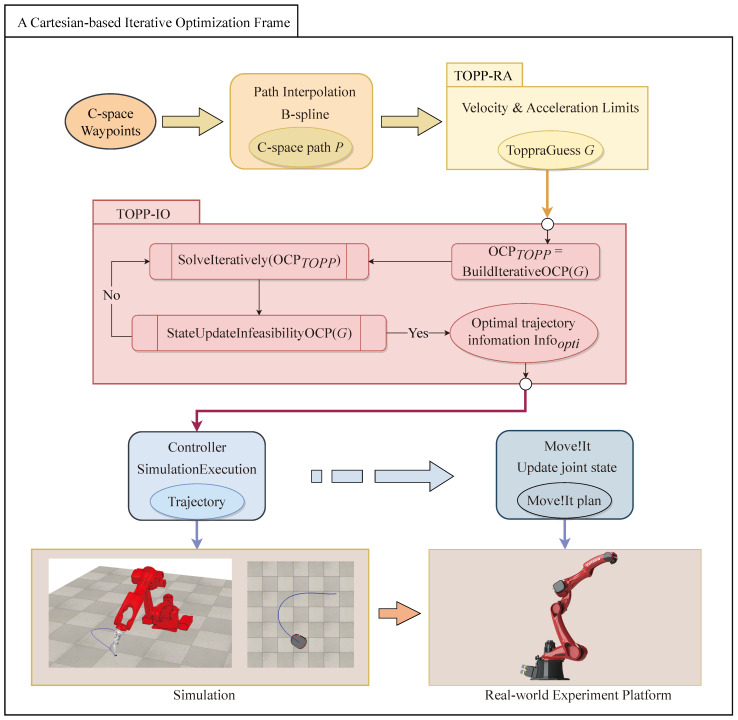
Architecture of implementation.

In this section, simulation experiments will be used to demonstrate the feasibility, performance, and generality of the proposed method, as well as an industrial robot real machine-verification experiment will be performed, which gives practical significance to the TOPP-IO algorithm. The proposed method is executed on Ubuntu using an Intel i7-7700HQ @ 2.80 GHz CPU and 16-GB RAM, and all optimization problems are solved using CasADi (CasAdi is an open-source software framework for nonlinear optimization and optimal control. It provides a flexible and efficient interface for constructing and solving various optimization problems, including trajectory optimization) [34]. We use the 6-DOF Firefox robot from SIASUN in both the simulation and the real world, in addition to the Pioneer P3-DX robot used in the simulation. The implementation of TOPP-IO was done in C++, and the required communication between systems for these experiments was established. Figure 1 illustrates the architecture of the implementation.

### 4.1. Experiment Settings

The joint and wheel velocity, acceleration, and jerk limits are presented in each experiment, respectively, which are critical factors for the safe and efficient operation of robotic systems. To assess the robustness and adaptability of our proposed algorithm, TOPP-IO, we conducted a series of experiments with varying jerk limits. Specifically, we evaluated the performance of TOPP-IO under four different jerk limits: 0.1×, 1×, 10×, and 100× the default value. The basic parameters for the iterative optimization are carefully selected to ensure the convergence and efficiency of the optimization process, which are listed in Table 2.

### 4.2. Comparison with TOPP-RA Method

**Figure 2:**
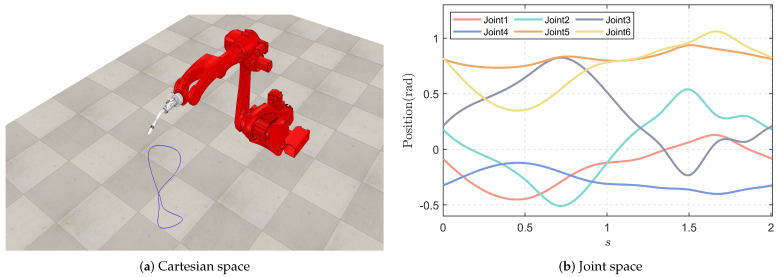
The geometric path on which this approach is implemented and tested.

This method is built and tested on the random geometric route depicted in Figure 2, subject to joint velocity, acceleration, and jerk limitations which are presented in Table 3. The simulation results are compared with those obtained from the CO algorithm (TOPP-RA) presented in [6] to demonstrate the effectiveness of the proposed strategy in controlling the acceleration surge caused by ignoring the jerk constraints.

**Figure 3:**
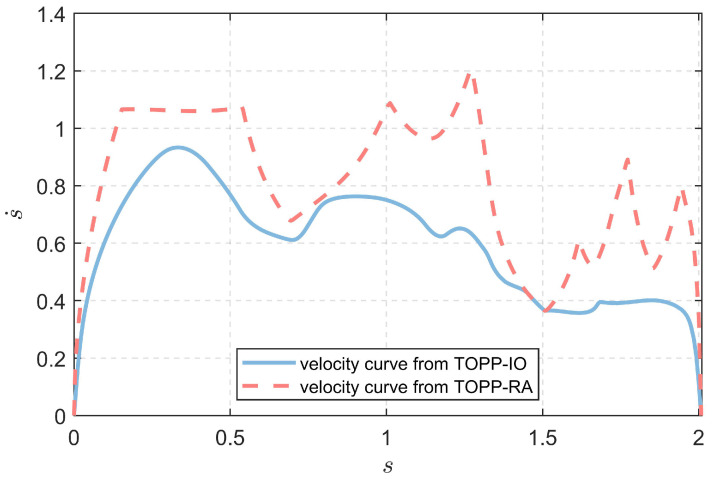
Comparison of the TOPP-RA resultant velocity curve without jerk limitations (red dashed line) and the one obtained from the proposed method with jerk limits (blue solid line).

**Figure 4:**
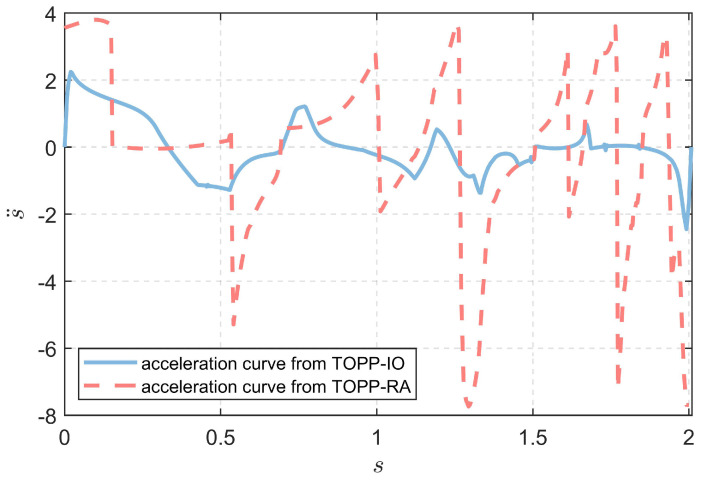
Comparison of the TOPP-RA resultant acceleration curve without jerk limitations (red dashed line) and the one obtained from the proposed method with jerk limits (blue solid line).

The results of the two approaches, TOPP-RA and TOPP-IO, in the $\left(s,\dot{s}\right)$ and $\left(s,\ddot{s}\right)$ phase planes are presented in Figure 3 and Figure 4, respectively. It can be observed from Figure 4 that TOPP-RA allows for steep slopes of acceleration due to the lack of restriction on jerk, leading to an abrupt shift in acceleration between neighboring path points. This sudden change in acceleration can be seen in the velocity curve of Figure 3, where there is no smooth transition between the acceleration and deceleration portions. Such abrupt changes in acceleration can result in jerky and unstable motion, which is not desirable in many real-world applications. To address this issue, TOPP-IO imposes explicit joint jerk limitations, leading to smoother acceleration profiles between neighboring path points. Figure 4 shows that the TOPP-IO method successfully restricts the acceleration mutation, preventing any abrupt changes in acceleration. Furthermore, the velocity curve of TOPP-IO in Figure 3 exhibits smoother transitions between the portions representing acceleration and deceleration, guaranteeing that the nearby segments will not violate the imposed restrictions.

**Figure 5:**
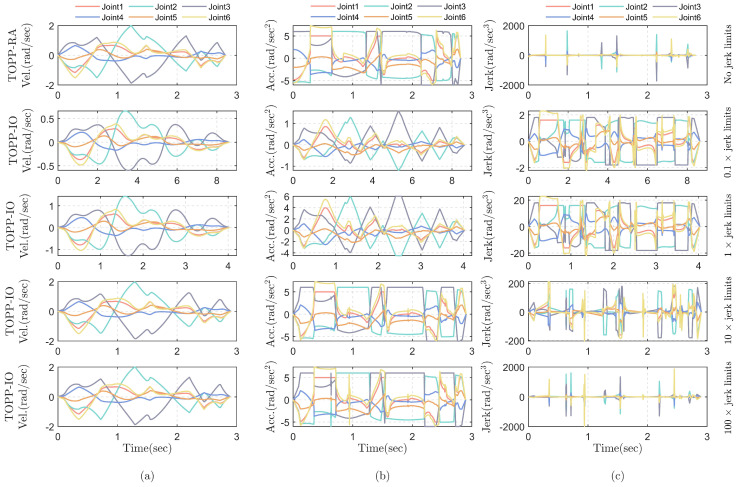
Velocity, acceleration, and jerk profiles for various methods and jerk restrictions. (**a**) Speed, (**b**) Acceleration, and (**c**) Jerk.

To further evaluate the performance of the two approaches, we compare their execution times in Table 4 and display the corresponding speed, acceleration, and jerk curves in Figure 5 for various jerk limits (100×, 10×, 1×, and 0.1×). In the TOPP-RA method, it is evident that the acceleration profiles are bang-bang, satisfying all joint second-order constraints.

With all third-order kinematic constraints, the jerk profiles are bang-bang in the TOPP-IO method, leading to smoother transitions between the portions representing acceleration and deceleration. Without joint jerk limits, the maximum acceleration is about 1638.4 rad/s${}^{3}$. As the jerk limit is decreased from “none” to 100× and 10× jerk limits, the execution time only slightly increases from 2.81067 s to 2.89393 s and 2.90326 s, respectively, and the smoothing effect of the speed profile is not immediately noticeable. The speed profile becomes smoother as the jerk limits approach 1× jerk limits. Notably, even when the jerk limit is set to 0.1× jerk limits, TOPP-IO can still produce a valid solution, albeit with an increased execution time.

### 4.3. Application on Mobile Robot

**Figure 6:**
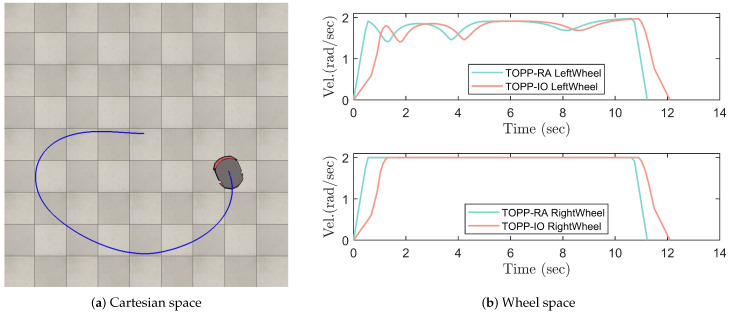
The ground path on which this approach is implemented and tested.

Our method applies not only to manipulators but also to a wide range of robots. To demonstrate its flexibility, we computed a ground trajectory for the Pioneer P3-DX, a diff-drive mobile robot. The wheel velocity, acceleration, and jerk limitations are presented in Table 5. Screenshots of the operational phase as well as the wheel speed curve in comparison to TOPP-RA are shown in Figure 6.

**Figure 7:**
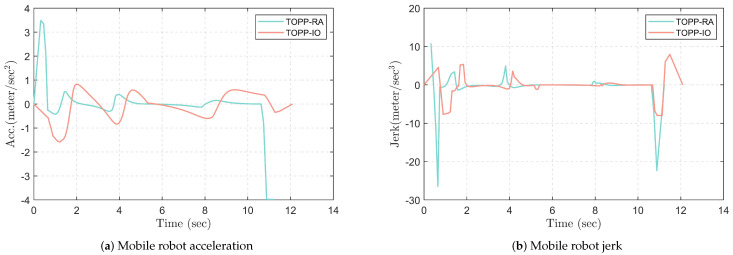
Comparing the resulting mobile robot acceleration (**a**) and jerk (**b**) from TOPP-RA and the ones obtained from the proposed approach.

The restrictions on wheel jerk and route jerk constraints have similar effects on controlling acceleration mutation. In this study, jerk restrictions were defined as wheel jerk constraints that effectively limit acceleration mutation in the route. The robot trajectories obtained from TOPP-RA and the proposed method are presented in Figure 7. Table 6 indicates that the maximum absolute values of the robot’s acceleration and jerk curves obtained from the proposed method are reduced by 60.28% and 69.82%, respectively, compared to those from TOPP-RA.

### 4.4. Real-World Experiments

**Figure 8:**
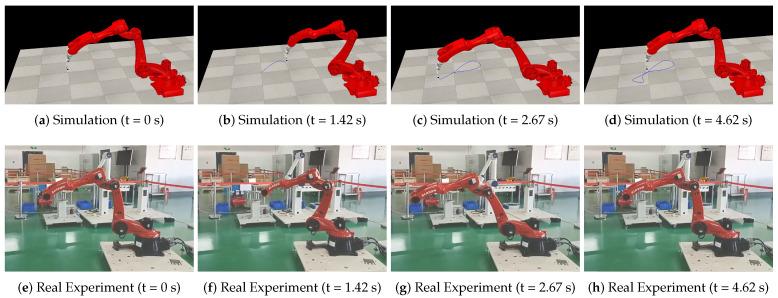
Real-world experiments (**e**–**h**) in accordance with the simulation (**a**–**d**).

In real-world experiments, we applied our method to the welding industry where the objective is to complete tasks as quickly, safely, and efficiently as possible. TOPP-IO succeeded in executing the assignment in a timely, safe, and stable manner. The running state of the Firefox robot in the actual world is shown in Figure 8 and is consistent with the simulation results.

**Figure 9:**
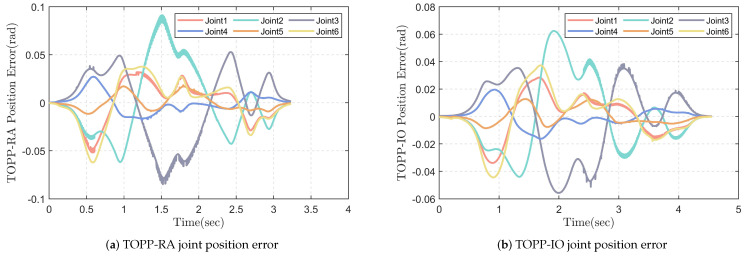
Comparing the resulting joint position error from TOPP-RA (**a**) and the ones obtained from proposed approach (**b**).

We performed both quantitative and qualitative analyses of our method’s performance during the actual operation process. Specifically, we analyzed the position error of each joint and examined the speed-tracking situation using joint1 as an example. Figure 9a shows the joint position error of the TOPP-RA method during actual operation, while Figure 9b displays the joint position error of the TOPP-IO method under the same path. In addition, Table 7 compares the performance of our TOPP-IO method with that of the TOPP-RA method. The results show that the average and maximum position errors of all joints in TOPP-IO have been reduced to different degrees during operation. The absolute values of the average position error and maximum position error have been reduced by about 29% and 27%, respectively, compared to the TOPP-RA method.

**Figure 10:**
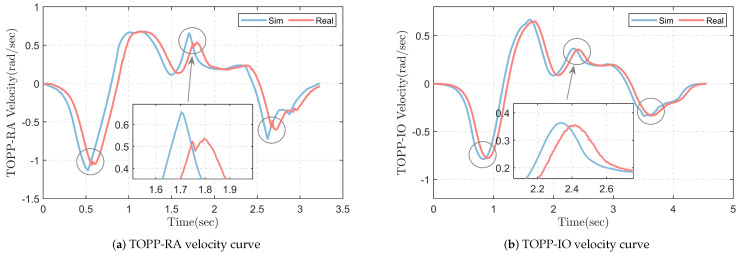
Comparing the resulting joint1 velocity curve from TOPP-RA (**a**) and the ones obtained from proposed approach (**b**). (The gray circle represented the snap-point).

To examine the speed-tracking situation, we used joint1 as an example. Figure 10a shows the speed-tracking of the TOPP-RA method during actual operation, while Figure 10b presents the speed curve of our TOPP-IO method, which considers the third-order constraint. Only the second-order constraint of joint space is considered, which leads to snap-point (represented by the gray circle), or the sudden change in joint acceleration, resulting in the inability to track the given speed on the actual physical robot. As shown in the zoomed-in section of Figure 10a, the snap-point causes fluctuations in the speed curve. In contrast, our TOPP-IO method eliminates the snap-point and enables smooth tracking of joint speed. Our method ensures a smooth trajectory and efficient, steady completion of the task while maintaining high speed.

## 5. Conclusions

In this paper, we develop a comprehensive and efficient iterative optimization framework for solving the TOTP problem with joint third-order constraints. The main contributions and results of this paper are as follows:

- The framework is constructed from the bottom up in the Cartesian coordinate system and can be applied to both manipulator and mobile robots;
- Our study has identified two main challenges in the framework: how to consistently represent the TOTP problem in the Cartesian space using the $\left(s,\dot{s}\right)$ phase plane, while imposing third-order kinematic constraints on each joint, and how to devise an efficient computational solution strategy that uses a constraint relaxation approach to simplify nonconvex constraints without violating them;
- We demonstrated the effectiveness of our proposed framework through both simulation and physical experiments. Compared to the TOPP-RA method, our approach effectively reduced the maximum absolute values of the robot’s jerk and the average absolute values of the position error over 60% and 29%, respectively. These are critical factors in ensuring smooth robotic velocity tracking and reducing impact during operation.

Our framework has a few limitations. First, we assume that the path of the end-effector in Cartesian space is predetermined. We use B-spline interpolation to generate continuous, smooth end-effector poses from the given path points. Second, our approach accepts suboptimal solutions when a feasible solution can be obtained at any point by interrupting the iterative optimization process.

Our future work can be divided into two main areas:

- First, we aim to extend our framework to handle both path planning and speed planning simultaneously, which will enable our method to generate feasible solutions more efficiently;
- Second, we plan to explore the potential of the constraint relaxation approaches and achieve real-time performance. Moreover, handling dynamic environments is a challenging and interesting area for future research.

## Figures and Tables

**Table 1:** A brief overview of related methods.

Methods		Calculate Switch Points	Optimization Objectives (Simple, Multiple)	Achieve Optimal Point	Planning Space	Constraint Order (Second-Order, Third-Order)
NI-based	[7,11,12]	Need	Simple	Yes	Joint/Cartesian	Second-order
	[8,9]	Need	Simple	Yes	Joint	Second-order
	[10,13]	Need	Simple	Yes	Joint	Third-order
CO-based	[6,17]	Not need	Simple	Yes	Joint	Third-order
	[14,15]	Not need	Multiple	Yes	Joint/Cartesian	Second-order
	[16]	Not need	Multiple	Yes	Joint/Cartesian	Third-order (Limit)
	[18]	Need	Simple	Yes	Joint	Third-order (Limit)
DP-based	[19,20]	Not need	Multiple	No	Joint	Third-order
	[21]	Not need	Multiple	No	Joint/Cartesian	Third-order
	[22]	Not need	Multiple	No	Joint	Second-order
	Ours	Not need	Multiple	Yes	Joint/Cartesian	Third-order

**Table 2:** Hyperparameter setting for iterative optimization.

Hyperparameter	Description	Value
$iter_{max}$	Maximum iteration number	5
$\omega_{soft0}$	$\omega_{soft}$ initial value	10${}^{5}$
α	Multiplier to enlarge $\omega_{soft}$	10
$e_{soft}$	Softened constraints tolerance	16

**Table 3:** Velocity, acceleration, and jerk limits of joints.

Limits	Joint1	Joint2	Joint3	Joint4	Joint5	Joint6
Vel. (rad/s)	2	2	2	4	4	4
Acc. (rad/s${}^{2}$)	5	6	6	12	12	12
Jerk (rad/s${}^{3}$)	16	16	18	20	28	28

**Table 4:** Execution time of different trajectory planning algorithms and jerk restrictions.

Method	TOPP-RA	TOPP-IO			
Jerk Limits (rad/s${}^{3}$)	-	100×	10×	1×	0.1×
t${}_{e}$ (s)	2.81067	2.89393	2.90326	4.02941	8.63447

**Table 5:** Velocity, acceleration, and jerk limits of wheels.

Limits	Wheel1	Wheel2
Vel. (rad/s)	2	2
Acc. (rad/s${}^{2}$)	4	4
Jerk (rad/s${}^{3}$)	8	8

**Table 6:** Comparing the maximum absolute value of the robot’s acceleration and jerk curves between the two approaches.

	Acceleration (m/s^2^)	Jerk (m/s^3^)
TOPP-RA	3.98086	26.4858
TOPP-IO	1.58118	7.9937
Degree of decline	60.28%	69.82%

**Table 7:** The absolute values of the average and maximum joint position error on different trajectory planning algorithms.

		Joint1	Joint2	Joint3	Joint4	Joint5	Joint6
**Average** **position error**	TOPP-RA (rad)	0.0160	0.0296	0.0293	0.0075	0.0066	0.0192
	TOPP-IO (rad)	0.0112	0.0205	0.0206	0.0053	0.0047	0.0135
	Degree of decline	30.13%	30.67%	29.81%	29.60%	29.65%	29.46%
**Maximum** **position error**	TOPP-RA (rad)	0.0518	0.0911	0.0849	0.0270	0.0183	0.0621
	TOPP-IO (rad)	0.0339	0.0624	0.0557	0.0196	0.0127	0.0444
	Degree of decline	34.63%	31.54%	34.36%	27.62%	30.36%	28.47%

## Data Availability

Not applicable.

## Abbreviations

The following abbreviations are used in this manuscript:

TOTP	time-optimal trajectory planning
TOPP	time-optimal path parameterization
NI	Numerical Integration
CO	Convex Optimization
DP	Dynamic Programming
DC	difference of convex
SCP	sequential convex programming
TOPP-RA	TOPP approach based on reachability analysis
TOPP-IO	TOPP approach based on iterative optimization
LP	linear programming
BA	bisection algorithm
NLP	nonlinear programming

## Appendix A. From Configuration Space to Cartesian Space

In this appendix, we introduce how to determine the position and attitude of the end-effector and their derivatives using the robot joint variables and their derivatives of each order, and then convert them into path parameter *s* for representation. This process involves forward kinematics, inverse kinematics and the derivatives of Jacobian matrix, which will be described in detail in the following subsections.

### Appendix A.1. Forward and Inverse Kinematics

ψ is defined as the screw coordinate of the spiral axis relative to the spatial coordinate system. Its Lie algebra representation is as follows:

(A1) $$se\left(3\right)=\left\{\psi =\left[\begin{array}{c} \rho \\ \phi \end{array}\right]\in R^{6},\rho \in R^{3},\phi \in so\left(3\right),\psi^{\wedge }=\left[\begin{array}{cc} \phi^{\wedge } & \rho \\ 0^{T} & 1 \end{array}\right]\in R^{4\times 4}\right\}$$

The Lie group corresponding to ψ can be expressed as

(A2) $$SE\left(3\right)=\left\{\Psi =\left[\begin{array}{cc} R & \varrho \\ 0^{T} & 1 \end{array}\right]\in R^{4\times 4},R\in SO\left(3\right),\varrho \in R^{3}\right\}$$

where R and ϱ separately denote the directions and positions of the rigid-body. The mapping between the Lie algebra ψ and the corresponding Lie group Ψ is given by:

(A3) $$\Psi =\exp\left(\psi^{\wedge }\right)=\left[\begin{array}{cc} \exp(\phi^{\wedge }) & \frac{\left(I-\exp\left(\phi^{\wedge }\right)\right)\phi^{\wedge }\rho +\phi \phi^{T}\rho }{{∥\phi ∥}^{2}}\\ 0^{T} & 1 \end{array}\right]$$

Consider a robot system with *n* degrees of freedom, whose configuration is represented by $q={\left[q_{1},q_{2},\cdots ,q_{n}\right]}^{T}\in R^{n}$. The forward kinematics model of the robot is determined as follows:

(A4) $$\Psi_{end}\left(q\right)=\prod_{i=1}^{n}\exp\left(\psi_{i}^{\wedge }q_{i}\right)\Psi_{S}=\left[\begin{array}{cc} R_{end} & \varrho_{end}\\ 0^{T} & 1 \end{array}\right]$$

where $\Psi_{S}$ represents the pose matrix of the end coordinate system relative to the space coordinate system when the robot is in the initial position; $\left(q_{i},\psi_{i}\right)$ denote the joint position and twist, respectively, of the *i*-th joint; $R_{end}$ and $\varrho_{end}$ separately denote the orientations and positions of the end-effector.

For a given robot model, it is possible to convert the end pose p expressed in Cartesian space to the corresponding joint values q in joint space using inverse kinematics. Similarly, the joint values q can be converted to the end pose p through forward kinematics. Thus, the reciprocal transformation between p and q can be achieved through these two transformations.

### Appendix A.2. Explicit Expressions of High-Order Jacobian Derivatives

Jacobian matrix in Formula (13) is as follows:

(A5) $$J=J_{p}\cdot J_{b}=\left[\begin{array}{cc} A^{-1}\left(r\right) & O\\ O & R_{sb}\left(r\right) \end{array}\right]\cdot \left[\begin{array}{cccc} \beta_{1}, & \beta_{2}, & \cdots , & \beta_{n} \end{array}\right]$$

where $J_{b}\left(q\right)\in R^{6\times n}$ represents the geometric Jacobian matrix; $r\in R^{3}$ represents the exponential coordinate of the axis angle, which is reflected in the last three lines of the p(s). In $J_{p}\in R^{6\times 6}$, $A\left(r\right)\in R^{3\times 3}$ and $R_{sb}\left(r\right)\in R^{3\times 3}$ can be expressed as:

(A6) $$\begin{array}{cc} A(r) & =I-\frac{1-\cos∥r∥}{{∥r∥}^{2}}r^{\wedge }+\frac{∥r∥-\sin∥r∥}{{∥r∥}^{3}}{\left(r^{\wedge }\right)}^{2},\\ R_{sb}\left(r\right) & =\exp(r^{\wedge }). \end{array}$$

Since r can be expressed by p(s), the first-order and second-order path parameter derivatives of $J_{p}$ are expressed as follows:

(A7) $$\begin{array}{cc} J_{p}^{\prime }\left(p\left(s\right)\right) & =\left[\begin{array}{cc} -A^{-1}\left(p\left(s\right)\right)\frac{dA(p(s))}{ds}A^{-1}\left(p\left(s\right)\right) & 0\\ 0^{T} & \frac{dR_{sb}}{ds} \end{array}\right],\\ J_{p}^{\prime \prime }\left(p\left(s\right)\right) & =\left[\begin{array}{cc} 2A^{-1}\frac{dA}{ds}A^{-1}\frac{dA}{ds}A^{-1}-A^{-1}\frac{d^{2}A}{ds^{2}}A^{-1}\; & 0\\ 0^{T} & \frac{d^{2}R_{sb}}{ds^{2}} \end{array}\right]. \end{array}$$

(1) First-order path parameter derivative $J^{\prime }$: The first-order derivative of Jacobian matrix J with respect to the path parameter *s* is as following:
  (A8) $$J=J_{p}^{\prime }\cdot J_{b}+J_{p}\cdot J_{b}^{\prime }$$
  The matrices $J_{p}$ and $J_{p}^{\prime }$ depend on the path parameter *s*, while $J_{b}$ is a function of the joint values q, which can be obtained by forward and inverse kinematics. Each column of $J_{b}\left(q\right)$ can be represented as an adjoint matrix, given by:
  (A9) $$\begin{array}{cc} \beta_{i} & =Ad_{\Psi_{i}}^{-1}\left(\psi_{i}\right)={\left(\Psi_{i}\psi_{i}^{\wedge }\Psi_{i}^{-1}\right)}^{\vee }\in R^{6},\mathrm{where}\\ \Psi_{i} & =\prod_{j=1}^{n}\exp\left(\psi_{j}^{\wedge }q_{j}\right)\Psi_{S}. \end{array}$$
  According to the chain rule of differentiation, the first-order derivative of the geometric Jacobian matrix with respect to the path parameter *s*, denoted as $J_{b}^{\prime }$, and its *i*-th column, denoted as $\beta_{i}^{\prime }$, can be expressed as:
  (A10) $$\begin{array}{cc} J_{b}^{\prime } & =\left[\begin{array}{cccc} \beta_{1}^{\prime }, & \beta_{2}^{\prime }, & \cdots , & \beta_{n}^{\prime } \end{array}\right],\\ \beta_{i}^{\prime } & =\sum_{j=1}^{n}\frac{\partial \beta_{i}}{\partial q_{j}}q_{j}^{\prime }\\ \; & =\left[\begin{array}{cccc} \frac{\partial \beta_{i}}{\partial q_{1}}, & \frac{\partial \beta_{i}}{\partial q_{2}}, & \cdots , & \frac{\partial \beta_{i}}{\partial q_{n}} \end{array}\right]q^{\prime }\\ \; & =\left[\begin{array}{cccc} \frac{\partial \beta_{i}}{\partial q_{1}}, & \frac{\partial \beta_{i}}{\partial q_{2}}, & \cdots , & \frac{\partial \beta_{i}}{\partial q_{n}} \end{array}\right]J^{-1}p^{\prime }. \end{array}$$
  In combination with the literature [35], $\frac{\partial \beta_{i}}{\partial q_{j}}$ can be calculated using $\left(\beta_{i},\beta_{j}\right)$ as follows:
  (A11) $$\frac{\partial \beta_{i}}{\partial q_{j}}=\left\{\begin{array}{cc} 0, & j<i\\ ad_{\beta_{j}}\left(\beta_{i}\right)={\left(\beta_{j}^{\wedge }\beta_{i}^{\wedge }-\beta_{i}^{\wedge }\beta_{j}^{\wedge }\right)}^{\vee }, & j\geq i \end{array}\right.$$
(2) Second-order path parameter derivative $J^{\prime \prime }$: The second-order derivative of the Jacobian matrix J with respect to the path parameter *s* is given by:
  (A12) $$J=J_{p}^{\prime \prime }\cdot J_{b}+J_{p}^{\prime }\cdot J_{b}^{\prime }+J_{p}\cdot J_{b}^{\prime \prime }$$
  According to the chain rule, the second-order path parameter derivative, $J_{b}^{\prime \prime }$, and its *i*-th column, $\beta_{i}^{\prime \prime }$, can be denoted as:
  (A13) $$\begin{array}{cc} J_{b}^{\prime \prime }= & \left[\begin{array}{cccc} \beta_{1}^{\prime \prime }, & \beta_{2}^{\prime \prime }, & \cdots , & \beta_{n}^{\prime \prime } \end{array}\right],\\ \beta_{i}^{\prime \prime }= & \frac{\partial }{\partial s}\left(\sum_{j=1}^{n}\frac{\partial \beta_{i}}{\partial q_{j}}q_{j}^{\prime }\right)\\ = & \sum_{j=1}^{n}\frac{\partial \beta_{i}}{\partial q_{j}}q_{j}^{\prime \prime }+\frac{\partial }{\partial s}\left(\sum_{j=1}^{n}\frac{\partial \beta_{i}}{\partial q_{j}}\right)q_{j}^{\prime }\\ = & \left[\begin{array}{cccc} \frac{\partial \beta_{i}}{\partial q_{1}}, & \frac{\partial \beta_{i}}{\partial q_{2}}, & \cdots , & \frac{\partial \beta_{i}}{\partial q_{n}} \end{array}\right]q^{\prime \prime }\\ \; & +\left[\begin{array}{cccc} \frac{\partial }{\partial s}\left(\frac{\partial \beta_{i}}{\partial q_{1}}\right), & \frac{\partial }{\partial s}\left(\frac{\partial \beta_{i}}{\partial q_{2}}\right), & \cdots , & \frac{\partial }{\partial s}\left(\frac{\partial \beta_{i}}{\partial q_{n}}\right) \end{array}\right]J^{-1}p^{\prime }. \end{array}$$
  where
  (A14) $$q^{\prime \prime }=J^{-1}\left(p^{\prime \prime }-J^{\prime }J^{-1}p^{\prime }\right)$$
  and
  (A15) $$\frac{\partial }{\partial s}\left(\frac{\partial \beta_{i}}{\partial q_{j}}\right)=\left\{\begin{array}{cc} 0, & j<i\\ ad_{\beta_{j}}\left(\beta_{i}^{\prime }\right)+ad_{\beta_{j}^{\prime }}\left(\beta_{i}\right), & j\geq i \end{array}\right.$$

Overall, this appendix establishes the forward kinematics of a robot using Lie theory and symbolically derives the first-order and second-order derivatives of the Jacobian matrix. Higher-order Jacobian matrices could be derived similarly. Furthermore, the inverse kinematics for a specific robot model can be easily obtained.

## References

[1] Mikolajczyk T. (2012). Manufacturing Using Robot. *Adv. Mater. Res.*
**2012**, *463*, 1643–1646. Proceedings of the Advanced Materials Research II.

[2] Oztemel E., Gursev S. (2020). Literature review of Industry 4.0 and related technologies. J. Intell. Manuf..

[3] Chiurazzi M., Alcaide J.O., Diodato A., Menciassi A., Ciuti G. (2023). Spherical Wrist Manipulator Local Planner for Redundant Tasks in Collaborative Environments. Sensors.

[4] Gasparetto A., Boscariol P., Lanzutti A., Vidoni R. (2012). Trajectory Planning in Robotics. Math. Comput. Sci..

[5] Zhang T., Zhang M., Zou Y. (2021). Time-optimal and Smooth Trajectory Planning for Robot Manipulators. Int. J. Control. Autom. Syst..

[6] Pham H., Pham Q.-C. (2018). A New Approach to Time-Optimal Path Parameterization Based on Reachability Analysis. IEEE Trans. Robot..

[7] Bobrow J.E., Dubowsky S., Gibson J.S. (1985). Time-Optimal Control of Robotic Manipulators Along Specified Paths. Int. J. Robot. Res..

[8] Kunz T., Stilman M. (2012). Time-optimal trajectory generation for path following with bounded acceleration and velocity. Robotics: Science and Systems VIII.

[9] Pham Q.C. (2014). A General, Fast, and Robust Implementation of the Time-Optimal Path Parameterization Algorithm. IEEE Trans. Robot..

[10] Pham H., Pham Q.C. On the structure of the time-optimal path parameterization problem with third-order constraints. Proceedings of the 2017 IEEE International Conference on Robotics and Automation (ICRA).

[11] Shen P., Zhang X., Fang Y. (2017). Essential Properties of Numerical Integration for Time-Optimal Path-Constrained Trajectory Planning. IEEE Robot. Autom. Lett..

[12] Shen P., Zhang X., Fang Y. (2018). Complete and Time-Optimal Path-Constrained Trajectory Planning With Torque and Velocity Constraints: Theory and Applications. IEEE/ASME Trans. Mechatronics.

[13] Lu L., Zhang J., Fuh J.Y.H., Han J., Wang H. (2020). Time-optimal tool motion planning with tool-tip kinematic constraints for robotic machining of sculptured surfaces. Robot.-Comput.-Integr. Manuf..

[14] Verscheure D., Demeulenäre B., Swevers J., De Schutter J., Diehl M. (2008). Practical time-optimal trajectory planning for robots: A convex optimization approach. IEEE Trans. Autom. Control..

[15] Xiao Y., Dong W., Du Z. A time-optimal trajectory planning approach based on calculation cost consideration. Proceedings of the 2012 IEEE International Conference on Mechatronics and Automation.

[16] Debrouwere F., Van Loock W., Pipeleers G., Dinh Q.T., Diehl M., De Schutter J., Swevers J. (2013). Time-Optimal Path Following for Robots With Convex-Concave Constraints Using Sequential Convex Programming. IEEE Trans. Robot..

[17] Nagy Á., Vajk I. (2019). Sequential Time-Optimal Path-Tracking Algorithm for Robots. IEEE Trans. Robot..

[18] Ma J.-w., Gao S., Yan H.-t., Lv Q., Hu G.-q. (2021). A new approach to time-optimal trajectory planning with torque and jerk limits for robot. Robot. Auton. Syst..

[19] Shin K., McKay N. (1986). A dynamic programming approach to trajectory planning of robotic manipulators. IEEE Trans. Autom. Control..

[20] Kaserer D., Gattringer H., Müller A. (2019). Nearly Optimal Path Following with Jerk and Torque Rate Limits Using Dynamic Programming. IEEE Trans. Robot..

[21] Kaserer D., Gattringer H., Müller A. (2020). Time Optimal Motion Planning and Admittance Control for Cooperative Grasping. IEEE Robot. Autom. Lett..

[22] Barnett E., Gosselin C. (2021). A Bisection Algorithm for Time-Optimal Trajectory Planning Along Fully Specified Paths. IEEE Trans. Robot..

[23] Faulwasser T., Findeisen R. (2016). Nonlinear Model Predictive Control for Constrained Output Path Following. IEEE Trans. Autom. Control..

[24] Consolini L., Locatelli M., Minari A., Piazzi A. (2017). An optimal complexity algorithm for minimum-time velocity planning. Syst. Control. Lett..

[25] Steinhauser A., Swevers J. (2018). An Efficient Iterative Learning Approach to Time-Optimal Path Tracking for Industrial Robots. IEEE Trans. Ind. Inform..

[26] Consolini L., Locatelli M., Minari A. (2022). A Sequential Algorithm for Jerk Limited Speed Planning. IEEE Trans. Autom. Sci. Eng..

[27] Petrone V., Ferrentino E., Chiacchio P. (2022). Time-Optimal Trajectory Planning With Interaction With the Environment. IEEE Robot. Autom. Lett..

[28] Yang Y., Xu H.z., Li S.h., Zhang L.l., Yao X.m. (2022). Time-optimal trajectory optimization of serial robotic manipulator with kinematic and dynamic limits based on improved particle swarm optimization. Int. J. Adv. Manuf. Technol..

[29] Singh S., Leu M.C. (1987). Optimal Trajectory Generation for Robotic Manipulators Using Dynamic Programming. J. Dyn. Syst. Meas. Control..

[30] Slotine J.J.E., Yang H.S. Improving the Efficiency of Time-Optimal Path-Following Algorithms. Proceedings of the 1988 American Control Conference.

[31] Consolini L., Locatelli M., Minari A., Nagy Á., Vajk I. (2019). Optimal Time-Complexity Speed Planning for Robot Manipulators. IEEE Trans. Robot..

[32] Li B., Ouyang Y., Li L., Zhang Y. (2022). Autonomous Driving on Curvy Roads Without Reliance on Frenet Frame: A Cartesian-Based Trajectory Planning Method. IEEE Trans. Intell. Transp. Syst..

[33] Guarino Lo Bianco C., Faroni M., Beschi M., Visioli A. (2022). A Predictive Technique for the Real-Time Trajectory Scaling Under High-Order Constraints. IEEE/ASME Trans. Mechatronics.

[34] Andersson J.A.E., Gillis J., Horn G., Rawlings J.B., Diehl M. (2019). CasADi—A software framework for nonlinear optimization and optimal control. Math. Program. Comput..

[35] Fu Z., Spyrakos-Papastavridis E., Lin Y.-H., Dai J.S. Analytical Expressions of Serial Manipulator Jacobians and their High-Order Derivatives based on Lie Theory. Proceedings of the 2020 IEEE International Conference on Robotics and Automation (ICRA).

